# Electroacupuncture Relieves Suppression of Autophagy in Interstitial Cells of Cajal of Diabetic Gastroparesis Rats

**DOI:** 10.1155/2020/7920715

**Published:** 2020-03-17

**Authors:** Xing Wei, Yaping Lin, Dongfeng Zhao, Xiaojuan Xiao, Qiao Chen, Siyi Chen, Yan Peng

**Affiliations:** ^1^The Domestic First-Class Discipline Construction Project of Chinese Medicine of Hunan University of Chinese Medicine, College of Acupuncture & Moxibustion and Tui-na, Hunan University of Chinese Medicine, Changsha 410000, China; ^2^Medical School, Hunan University of Chinese Medicine, Changsha 410000, China

## Abstract

**Background:**

The incidence of diabetic gastroparesis (DGP) is mainly blamed to abnormity of interstitial cells of Cajal (ICCs). Autophagy could degrade damaged proteins and organelles to keep intracellular homeostasis, and it could directly influence structure and number of cells. In this study, we aimed to figure out the relationship between DGP and autophagy of ICCs.

**Methods:**

Sixty Sprague-Dawley (SD) rats were randomly divided into normal control group (NC, 10) and modeling group (50). Rats in the modeling group were injected 2% streptozotocin (STZ) and fed with high-glucose and high-fat diet for 8 weeks in order to establish DGP rat model. After modeling, 30 successfully modeled rats were randomly selected and separated into diabetic gastroparesis group (DGP, 10), GDP rats with electroacupuncture group (EA, 10), and GDP rats with metoclopramide group (MP, 10). When the intervention was completed, blood glucose was measured by ONE TOUCH glucometer and gastrointestinal propulsive rate was detected through measuring optical density. Autophagosomes were observed under transmission electron microscope (TEM). The expression of LC3 protein and P62 protein was measured by Western blot. When ICCs were transfected with GFP-RFP-LC3 plasmid, autophagy flux was observed by laser scanning confocal microscope.

**Results:**

(1) After intervention, compared with blood glucose of rats in the NC group, all of the DGP, EA, and MP groups were remarkably increased (*P* < 0.01); compared with the DGP group, the blood glucose of the EA and MP groups was decreased greatly (*P* < 0.01); compared with the DGP group, the blood glucose of the EA and MP groups was decreased greatly (*P* < 0.01); compared with the DGP group, the blood glucose of the EA and MP groups was decreased greatly (*P* < 0.01); compared with the DGP group, the blood glucose of the EA and MP groups was decreased greatly (*P* < 0.01); compared with the DGP group, the blood glucose of the EA and MP groups was decreased greatly (*P* < 0.01); compared with the DGP group, the blood glucose of the EA and MP groups was decreased greatly (*P* < 0.01); compared with the DGP group, the blood glucose of the EA and MP groups was decreased greatly (*P* < 0.01); compared with the DGP group, the blood glucose of the EA and MP groups was decreased greatly (*P* < 0.01); compared with the DGP group, the blood glucose of the EA and MP groups was decreased greatly (*P* < 0.01); compared with the DGP group, the blood glucose of the EA and MP groups was decreased greatly (*P* < 0.01); compared with the DGP group, the blood glucose of the EA and MP groups was decreased greatly (*P* < 0.01); compared with the DGP group, the blood glucose of the EA and MP groups was decreased greatly (*P* < 0.01); compared with the DGP group, the blood glucose of the EA and MP groups was decreased greatly (*P* < 0.01); compared with the DGP group, the blood glucose of the EA and MP groups was decreased greatly (*P* < 0.01); compared with the DGP group, the blood glucose of the EA and MP groups was decreased greatly (*P* < 0.01). (2) Compared with gastrointestinal propulsive rate of rats in the NC group, no matter gastric emptying rate or intestinal propulsive rate, the EA and MP groups were significantly reduced (*P* < 0.01); compared with the NC group, gastric emptying rate and intestinal propulsive rate in the EA group were obviously decreased (*P* < 0.05, *P* < 0.01); compared with the DGP group, the EA and MP groups were increased significantly (*P* < 0.01). (3) Compared with the NC group, intensity of RFP and GFP in the DGP group was obviously increased (*P* < 0.05, *P* < 0.01), in other words, the DGP group accompanying suppression of autophagy; compared with the DGP group, intensity of RFP and GFP in the EA group was decreased significantly (*P* < 0.05, *P* < 0.01). (4) There was no autophagosome in the NC group, and an autophagosome existed in the DGP group. Both EA and MP groups found autophagy. (5) When coming to LC3 II/LC3 I, compared with the NC group, the ratio was enhanced in the DGP and EA groups (*P* < 0.01, *P* < 0.05); compared with the DGP group, LC3 II/LC3 I was dramatically decreased in the MP and EA groups (*P* < 0.01). (6) As the substrate of degradation, the expression of P62 in the other three groups was significantly increased (*P* < 0.01) compared with the NC group; compared with the DGP group, the amount of P62 in the EA and MP groups was reduced greatly (*P* < 0.01).

**Conclusion:**

The impaired autophagy flux in ICCs is the pathological basis of diabetic gastroparesis, blaming to fusion dysfunction of autophagosome and lysosome and electroacupuncture (EA) could ease the suppression of autophagy to improve gastric motility.

## 1. Background

Diabetic gastroparesis (DGP) is a common complication of long-lasting poorly controlled diabetes, showing a delay in gastric emptying without any mechanical obstruction [1, 2]. Symptoms associated with gastroparesis include nausea, vomiting, bloating, early satiety, abdominal pain, and fullness. It is reported 76% of outpatients with diabetes have at least one gastrointestinal (GI) symptom [3] and females have higher incidence than males [4]. Interstitial cells of Cajal (ICCs) are the pacemaker of slow waves, initiating contractions by triggering rhythmical electrical activity [5, 6]. Abnormality of ICCs is an important factor of DGP, and ICCs are the therapeutic targets of DGP [7]. More and more findings have suggested that the structural and quantitative abnormalities of ICC are the key to GI diseases [8–10]. Autophagy is a highly conserved approach to degrade and recycle organelles and proteins in eukaryotes [11], so it plays an important role in cell survival and homeostasis. Appropriate autophagy is protective for cells lacking of nutrition; however, excessive autophagy may induce cells type II programmed cell death. Study has demonstrated that high glucose inhibited autophagy in diabetic heart diseases [12], but there are no relevant reports focusing on the role of autophagy in DGP.

The commonly used treatment for DGP is metoclopramide (MP), but its effect is not good enough in long term, prone to drug resistance, such as dry mouth and dizziness [13]. In recent years, acupuncture treatment for gastrointestinal dyspepsia has attracted more and more attention from scholars. Clinical studies have shown that acupuncture has good effects on functional dyspepsia [14] and postoperative gastrointestinal dysfunction [15]. Our team has confirmed electroacupuncture (EA) on Zusanli (ST36), Liangmen (ST21), and Sanyinjiao (SP6) was effective in the treatment of DGP. On the one hand, EA reduced the level of blood glucose and improved gastrointestinal motility and constitutional symptoms. On the other hand, EA improved the ultrastructure of ICC, adjusted the upstream regulation factors of ICCs, such as SCF, INS, IGF 1, and Ghrelin [16–19], and enhanced the current of Ca^2+^-activated Cl^−^ channels in ICCs [20].

Based on the above studies, we hypothesized that improvement of the pace making function of ICC by EA is related to the regulation of autophagy. Therefore, this study established model of DGP in rats and observed autophagy in ICCs through dual fluoresce mRFP-EGFP-LC3. In the study, we aimed at figuring out the role of autophagy in DGP and the underlying mechanism of EA in treating DGP.

## 2. Methods

### 2.1. Ethics Statement

This study was carried out strictly in accordance with the recommendations in the Guide for the Care and Use of Laboratory Animals of the National Institutes of Health, with all operations approved by the Ethics Committee for Animal Research Studies at the Hunan SJA Laboratory Animal (Ethical number: IACUC-SJA18081).

### 2.2. Animals

Sixty Sprague-Dawley rats weighing 220–240 g, half male and half female, were provided by Hunan SJA Laboratory Animal (certificate number: scxk (Xiang) 2016-0002). The rats were housed at the Experimental Animal Center of Hunan SJA Laboratory Animal, with the temperature at 20–22°C, relative humidity of 65%–70%, and free access to water. Following adaptation for 1 week, all the rats were randomly allocated to the normal control group (NC, 10) and modeling group (50). After modeling, 30 successfully modeled rats were randomly selected and separated into the diabetic gastroparesis group (DGP, 10), GDP rats with the electroacupuncture group (EA, 10), and GDP rats with the metoclopramide group (MP, 10).

### 2.3. Animal Models

After fasting for 12 h, rats in the modeling group were intraperitoneally injected with a single dose of 2% streptozotocin (STZ, Sigma, S0130-1G, USA) quickly (55 mg/Kg). STZ was dissolved in 0.1 mmol/L sodium citrate buffer (Guangzhou Chemical Reagent Factory, Guangzhou, China) to prepare a 2% solution (4°C, pH 4.5). The rats in the NC group were also injected with citrate sodium citrate buffer. Seventy-two hours later, the blood glucose of tail vein was measured by blood glucose meter (ONE TOUCH UltraVue, Johnson and Johnson, USA). If blood glucose was ≥16.7 mmol/L and persisted at this level for 2 weeks, it was considered as successful model. A high-glucose and high-fat diet (regular diet : lard stearin : sucrose : milk powder : egg = 58 : 15 : 20 : 5 : 2) was then fed to model rats irregularly (morning in odd days and afternoon in even days) for 8 weeks.

In spite of blood glucose level, there were significant differences in GI propulsive indicators, such as gastric emptying rate and intestinal propulsion rate, between the model and the control group rats. In terms of comparing the rat injected with STZ to normal rats, *P* < 0.05 was considered that the DGP model was successfully established. In addition, characteristics of stool and fur color were also important reference indexes. In fact, 43 rats met molding standards. Therefore, 30 rats were randomly selected from 43 rats and separated into DGP group, EA group, and MP group.

### 2.4. Treatment Method

Rats in the NC group and the DGP group were tied up with string and administrated with 0.9% normal saline. Rats in the MP group were bond for 20 min and intragastrically administered 1.7% of MP (Shanxi Yunpeng Pharmaceutical Co. Ltd, China), 1.0 ml/100 g, once a day, 15 days. Rats in the EA group received intragastric administration of 0.9% normal saline and acupunctured on Zusanli (ST36), Liangmen (ST21), and Sanyinjiao (SP6). Zusanli (ST 36): it is posterolateral to knee joint and about 5 mm away from the capitulum fibulae. Liangmen (ST 21): tummy-button is considered the junction of the upper 3/4 and lower 1/4 of the connection line between the upper edge of the sternum and external genitalia. Liangmen (ST 21) is away from the midpoint of the connection line between xiphisternal synchondrosis and the tummy-button to the midpoint of the medioclavicular line. Sanyinjiao (SP 6): it is vertically 10 mm above medial ankle tip of hind legs. The selection method of acupoint was based on the Experimental Animal Acupoint Map [21]. EA was performed with EA apparatus (SDZ-II, Suzhou Medical Appliance Factory Co., Ltd., China) with sparse-dense wave (sparse wave 10 Hz, dense wave 50 Hz) for 15 days. The electric current was set at 2 mA, 20 min each time, once a day. The electric current was ensured according to slightly trembling of the lower limbs. After intervention for 15 days, rats were tested for gastric emptying, specimen collection, isolation, and culture of primary ICCs.

### 2.5. Gastric Emptying Rate and Intestinal Propulsive Ratio

After overnight deprivation of food, all rats received 2 ml phenol red solution (50 mg/dL, Tianjin Guangfu Fine Chemical Research Institute, China) administered by gavage. Twenty minutes later, rats were anesthetized by isoflurane (inducing concentration: 3%, maintaining concentration: 2%, R510-22, RWD, China) and underwent spinal dislocation, followed by a laparotomy and stomach was removed carefully after ligation at the cardia and pyloric. Stomach was cut along greater curvature, its contents were poured into a beaker, and it was washed with 20 ml 0.9% normal saline in the beaker. Then 20 ml NaOH solution (0.5 mol/L) and 0.5 ml 20% trichloroacetic acid were poured into the beaker. The absorbance of the supernatant is read at 560 nm with spectrophotometer (UV1800, Chengdu Shimadzu Equipment Co., Ltd., China) after centrifugation (3500 r/min, 10 min). The absorbance could reflect the amount of phenol red remaining in the stomach. Standard phenol red solution was prepared by mixing 2 mL phenol red solution (50 mg/dL) with 18 mL distilled water, 20 mL NaOH (0.5 mol/L), 4 mL 20% trichloroacetic acid, and lastly its absorbance was measured. The rate of gastric emptying was calculated according to the following formula: gastric emptying (%) = (1−*X*/*Y*) × 100%. *X* represents absorbance of phenol red collected from the stomach 20 min after gavage with phenol red. *Y* represents absorbance of standard phenol red solution. At the same time, intestinal propulsive rate was quickly measured after laparotomy. Whole small intestine was cut gently and was straighten on ice, and the length of small intestine and propelling length of the phenol red were measured. After cutting small intestine and dripping NaOH (0.5 mol/L), the place small intestine turning purple served as the propelling distance of the phenol red in the intestine. Intestinal propulsive rate = propelling length of the phenol red/total length of small intestine ×100%.

### 2.6. Isolation and Identification of ICCs

The gastric antrum tissues were quickly cut and the blood vessels were carefully separated after 3 washes with D-Hanks (4°C). The specimens of antrum were rinsed with 10 ml D-hank's solution containing 1% Penicillin-Streptomycin (Gibco, 15140–122, USA), and the gastric mucosa layer was stripped off. Gastric antrum tissues were cut and crushed into 1*∗*1*∗*1 mm^3^, and type II collagenase digestive solution (pH 7.0, type II collagenase 1.3 mg/ml, Sigma C7806, USA) was added, digested at 37°C for 30 min, and centrifuged at 1500 r/min for 5 min. And supernatant was discarded, and pellets were resuspended and passed through cell strainer. The cells were inoculated into a six-pore plate with DMEM/F12 (Sigma-Aldrich, USA) and then put into the incubator for culture under the condition of 37°C, 5% CO_2_. After 24 h, medium was replaced by DMEM/F12 medium without penicillin and streptomycin. After that, the fluid was changed every 3 days, and the growth of the cells was observed under an inverted microscope. In order to determine whether the cultured cells are ICC or not, the internationally accepted C-kit immunofluorescence was used for culture. The medium was poured out and rinsed with 0.01 mol/L PBS for 3 times, 5 min each time, and fixed with 4% paraformaldehyde solution at room temperature for 20 min. PBS rinse was performed 3 times, 5 min each time, and 5% goat serum (Sigma, G6767, USA) was sealed at room temperature for 30 min. Then 3 mL of PE labeled rabbit anti-rat C-kit monoclonal antibody (diluted with 0.01 mol/L PBS at 1 : 100, eBioscience, CD117, USA) was added, placed in a 37°C environment for 30 min, and then placed in a 4°C refrigerator overnight. The next day, the cells were taken out and left at 37°C for 30 min and then bleached with 0.01 mol/L PBS for 3 times, 5 min each time. The cells were observed and photographed under a fluorescence microscope.

### 2.7. Plasmid Transfection and Autophagy Flux

**Figure 1:**
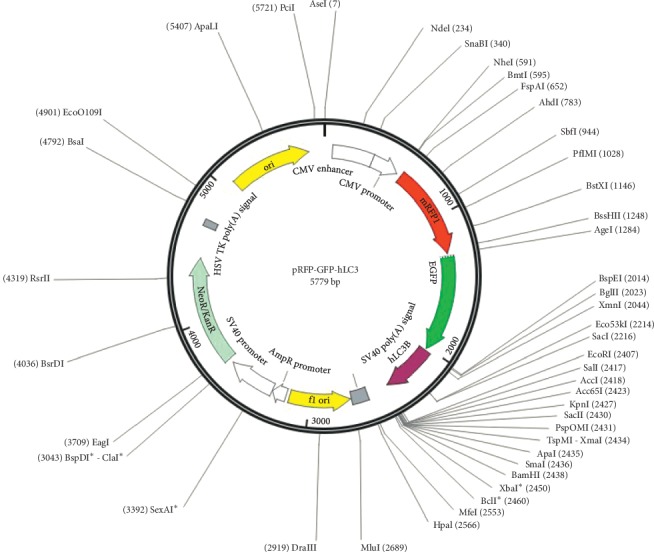
GFP-RFP-LC3 plasmid.

When the growth density of P2 of ICC was up to 60%–70% in six-well plates, the plasmid was transfected with Lipofectamine 2000 (Figure 1), according to the instructions of transfection reagent. And the culture medium containing transfection compound was replaced with culture medium after transfected for 6 h. Cells of logarithmic growth stage were inoculated on 6-well plates with a cell density of 2 × 10^5^/well. The complete medium was DMEM-F12 with 10% FBS (SH30084, Hyclone, USA) and 5 ng/ml stem cell factor (SCF, 400-22, USA). After transfected for 24 h, the cells were observed with a laser confocal microscope, photographed, and preserved. Five fields were randomly selected in each group and fluorescence intensity of each channel was measured.

### 2.8. Autophagy under Transmission Electron Microscope (TEM)

When gastric antrum tissues (1 mm × 1 mm × 1 mm) were obtained, the specimens were quickly put into fixation solution of electron microscope at 4°C for 2–4 h. Samples were rinsed 3 times with the 0.1 M phosphate buffer, each time for 15 min. And they were fixed in 1% osmium −0.1 M phosphate buffer (pH 7.4) at room temperature (20°C) for 2 h. Specimens were rinsed 3 times with phosphate buffer, each time for 15 min. The tissues were dehydrated in turn by 50%-70%-80%-90%-100%–100% alcohol-100% acetone-100% acetone, each time for 15 min. The samples were sliced after infiltration and embedding and dried overnight at room temperature. After the tissue was sliced (60–80 nm), the tissue was double-stained with uranium and lead (2% uranium acetate saturated alcohol solution and lead citrate, respectively, for 15 min) and dried overnight at room temperature. Then observed under TEM, images were collected and analyzed.

### 2.9. Western Blotting

The cells were washed twice with PBS, scraped with cell scraper, and centrifuged at 13000×g. The cell lysate buffer containing protease inhibitor was added into the cell precipitate and the cells were resuspended and allowed to lysate on ice for 10 min. Protein quantification was performed by BCA. Protein electrophoresis buffer was added and boiled at 100°C for 10 min. After electrical transfer to PVDF film, 5% skim milk was sealed for 1 h. LC3, p62, and *β*-actin antibodies were added and diluted at 1 : 1 000 and incubated overnight at 4°C. Followed with three washes of TBST, diluted secondary antibody was added and incubated at room temperature for 30 min. After three washes of TBST, the bands were visualized by chemical reaction with enhanced chemiluminescence agent. Protein relative expression was expressed by target protein *β*-actin.

### 2.10. Statistical Analysis

All data were analyzed by SPSS 22.0 software (SPSS, Inc., Chicago, IL, USA). Data were presented as the mean ± standard deviation. The comparisons among different groups were performed using the analysis of variance (ANOVA) and *t*-test. *P* < 0.05 was taken as a statistically significant difference.

## 3. Results

### 3.1. Blood Glucose

**Figure 2:**
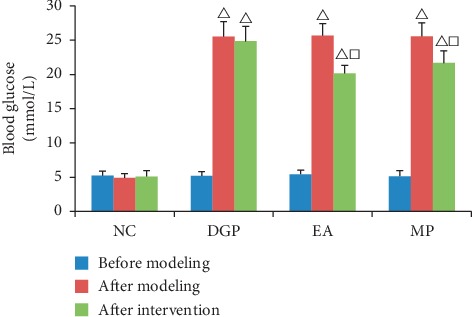
Blood glucose level. *P*^Δ^ < 0.01 versus rats in the NC group, *P*^□^ < 0.01 versus rats in the DGP group.

After modeling (Figure 2), except the NC group, blood glucose obviously increased in the other three groups (*P* < 0.01). When finishing intervention, compared with rats in the DGP group, blood glucose significantly decreased in the EA and MP groups (*P* < 0.01). However, there was still a large gap between the above two groups and the NC group.

### 3.2. Gastrointestinal Propulsive Rate

**Figure 3:**
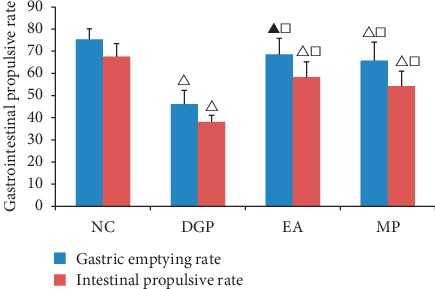
Gastrointestinal propulsive rate. ^Δ^*P* < 0.01 and *p*^▲^ < 0.05 versus rats in the NC group, *P*^□^ < 0.01 versus rats in the DGP group.

As seen in Figure 3, there was a sharp drop in the DGP group, no matter gastric emptying rate or intestinal propulsive rate (*P* < 0.01). It suggested that successful models had been accomplished. When compared with the DGP group, gastrointestinal propulsive rate in the EA and MP groups greatly increased. The EA group and MP group were comparable on gastrointestinal propulsive rate. Besides, it seemed electroacupuncture had a better effect on the stomach than on the small intestine.

### 3.3. Identification of ICCs

**Figure 4:**
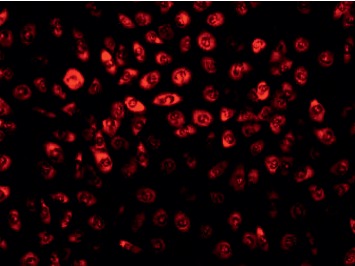
Identification of ICC through immunofluorescence.

After cultured for 2 weeks, cells were identified by immunofluorescence. Under the fluorescence microscope, cells showing red fluorescence were considered as C-kit-positive (Figure 4). After analyzing immunophenotype, we could confirm C-kit-positive cells were ICCs instead of smooth muscle cells (SMCs). In addition, in accordance with cell morphology, mast cells characterized by round cell and small nucleus were excluded.

### 3.4. Autophagy Flux

**Figure 5:**
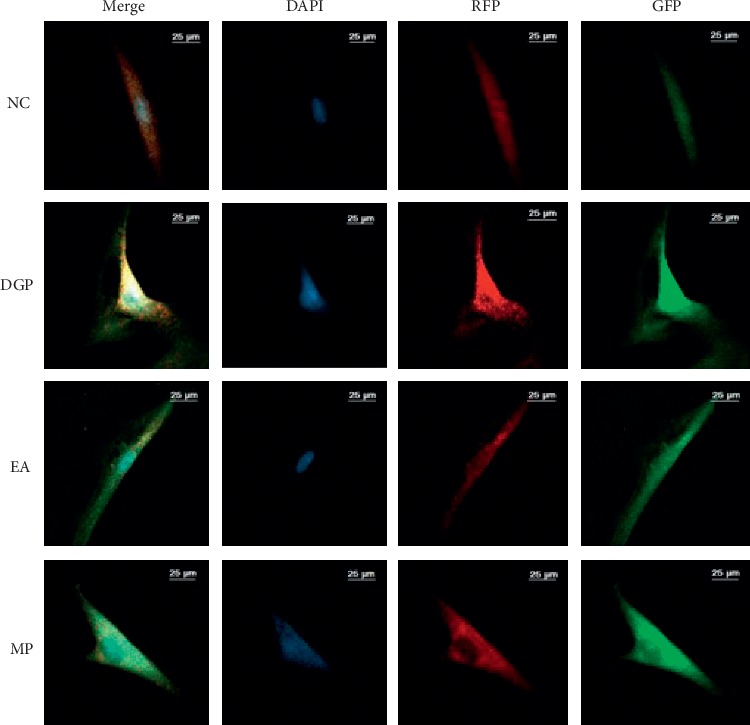
Autophagy of ICCs detected by laser scanning confocal microscope. Green fluorescent spots (GFP protein) represented autophagosomes that did not fuse with lysosomes, and red spots (RFP protein) were autophagolysosome. When red and green fluorescence fused, they could be shown as yellow fluorescence. The blue fluorescence was nucleus, dyed with DAPI.

**Figure 6:**
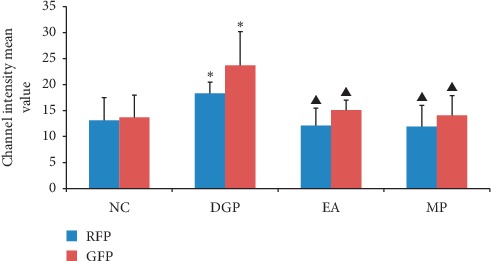
Intensity mean value of RFP and GFP. RFP: *P*^*∗*^ < 0.05 (*P*=0.041) versus rats in the NC group; *P*^▲^ < 0.05 (EA: *P*=0.017; MP: *P*=0.041) versus rats in the DGP group. GFP: *P*^*∗*^ < 0.01 (*P*=0.002) versus rats in the NC group; *P*^▲^ ≤ 0.01 (EA: *P*=0.007; MP: *P*=0.003) versus rats in the DGP group.

GFP (green fluorescence) was quenched in the acidic environment of autophagolysosome, representing autophagosomes that had not fused with lysosomes. RFP (red fluorescence) was resistant to acid and existed in all stages of autophagy, making autophagolysosome showing red. When the green and red fluorescence existed at the same time, it was yellow. If the yellow fluorescence was significantly enhanced, there were two possibilities. One possibility was induced autophagy; the other was impaired autophagy flux. As seen from Figures 5 and 6, the intensity of yellow fluorescence in the DGP group was enhanced while that in the EA group and the MP group was weakened. The specific situation should be combined with the subsequent results to draw a conclusion.

### 3.5. Autophagy under Transmission Electron Microscope

**Figure 7:**
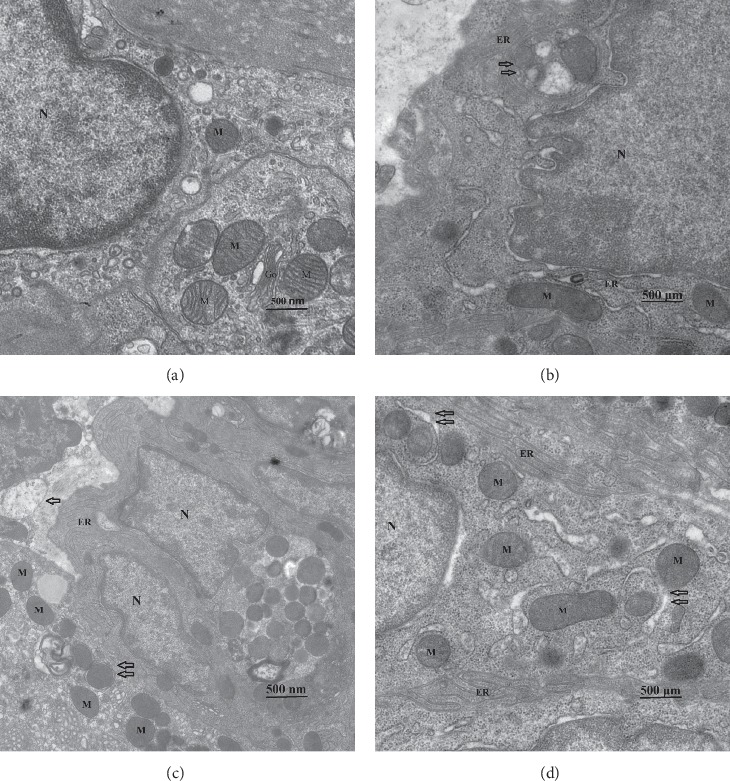
Autophagy under transmission electron microscope (TEM). (b) Autophagosome (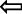), lipofuscin (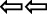); (c) autophagolysosome (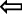), autophagosomes (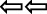); (d) endoplasmic reticulum (ER) extended and formed autophagosomes, and the mitochondria were wrapped (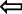). (a) NC. (b) DGP. (c) EA. (d) MP.

No autophagosome was found in the NC group, and there was abundant mitochondrion with clear structure. There was an autophagosome in the DGP group. One autophagosome and two autophagolysosomes were observed in the EA group. Two phagophores formed by endoplasmic reticulum (ER) were observed in the MP group, extending and wrapping mitochondrion, marking the beginning of autophagy (Figure 7).

### 3.6. Expression of LC3 and P62

**Figure 8:**
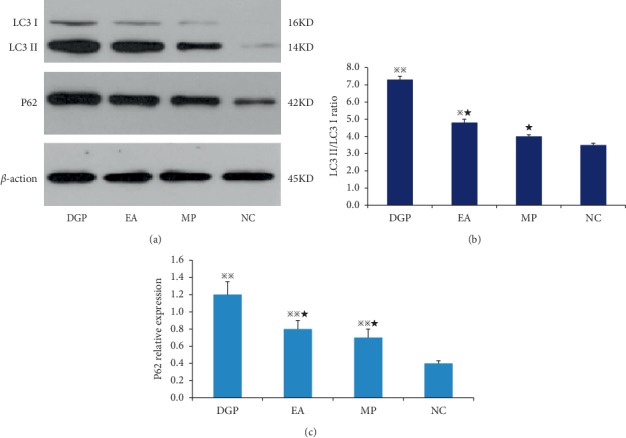
(a) The relative expression of P62 protein and LC3. There were significant differences in the NC, DGP, and intervention groups (EA and MP) on the ratio of LC3 II/LC3 I. (b)*P*^∗∗^ < 0.01, *P*^*∗*^ < 0.05 versus the NC group, *P*^★^ < 0.01 versus the DGP group. (c) The ratio of LC3II/LC3I in the DGP group significantly increased, and the EA group and the MP group were comparable (*P*=0.165). *P*^∗∗^ < 0.01 versus the NC group; *P*^★^ < 0.01 versus the DGP group. The relative expression of P62 protein and LC3 II/LC3 I in the DGP group were consist, obviously increasing. EA had the same effect as MP (*P*=0.273).

After analyzing Western blotting (Figure 8), we could clearly see LC3 II/LC3 I of the DGP group was apparently higher than the NC group, and the EA group was higher than the MP group. At the same time, we detected the relative expression of substrate of autophagy-P62. The content of P62 increased when autophagy flux was suppressed. The expression of P62 protein in the DGP group was the highest, the second was the MP and EA groups, and the NC group was the least. It reminded us that autophagy in ICC was inhibited on the condition of DGP.

## 4. Discussion

In the present study, we found EA could significantly reduce the level of blood glucose and improve gastrointestinal motility in DGP rats, which is consistent with previous studies [22–24]. More importantly, we found there was impaired autophagy flux in DGP rats, mainly focusing on the fusion disorder of autophagosomes and lysosomes. And the impaired autophagy flux could be alleviated by EA.

Autophagy widely exists in all eukaryotic cells, a lysosome-depended degradation pathway, transporting intracellular proteins and damaged organelles to the lysosomes to degrade. Autophagy maintains normal function and homeostasis, which can create a stable environment for the survival of the cell itself. Impaired autophagy flux influences the number and structure of cells. Many studies indicated that there was suppression of autophagy in diabetic heart disease and diabetic kidney disease [12, 25]. When it comes to DGP, more attention has been paid to organelle degeneration and loose gap junction in ICCs. Few reports focused on the role that autophagy played in DGP and its regulatory mechanism during EA treatment. In order to explore the problem above, we adopted an RFP-GFP-LC3 autophagy indicator system, the ratio of LC3 II/I and p62 combined with TEM. The greatest advantage of RFP-GFP-LC3 dual fluorescence autophagy indicator system is that it can visually judge changes in autophagy activity and autophagy flux without additional drug intervention. After induction of autophagy, the fusion-expressed fluorescent protein-LC3 protein is anchored on the membrane of autophagosome and fuses with lysosomes together with autophagosome. Among them, GFP protein is an acid-sensitive protein, and the acidic environment in lysosome can cause GFP fluorescence signal quenching, while RFP is a stable fluorescent protein, which is not influenced by the acidic environment. After entering the second stage, autophagosomes fuse with lysosomes to form autophagolysosome. Due to the acidic environment inside the lysosome, the PH decreases and GFP is quenching. The autophagosomes and autophagolysosomes were labeled with green and red, respectively. If autophagy flux runs smoothly, the green fluorescence would degrade in the acidic environment of the autophagolysosome, showing red fluorescence. When the fusion of autophagosomes and lysosomes was suppressed, the autophagy flux was blocked. Therefore, the green fluorescence was not quenched, and the fusion with the red fluorescence formed yellow fluorescence. In this study, the intensity of yellow fluorescence and green fluorescence in the DGP group was significantly enhanced. If the yellow fluorescence was significantly enhanced, there were two possibilities. One possibility was induced autophagy; the other was impaired autophagy flux. Combined with the results of LC3 II/LC3 I and P62, it was obvious to find the latter was the result. In other words, there was inhibited autophagy flux in DGP rats and the autophagosomes failed to fuse with lysosomes. Therefore, abnormalities of ICCs in DGP resulted from suppression of autophagy flux which contributed to the inability to degrade and recycle damaged organelles and proteins. After the intervention of EA and MP, the intensity of green fluorescence was significantly decreased, suggesting the inhibition of autophagy was effectively relieved. It was consistent with the enhanced gastric motility in the EA and MP groups.

Transmission electron microscopy (TEM) is the most direct and classical method to observe autophagy. Autophagy detected by TEM is mainly based on the identification of structures of autophagosomes and autophagolysosomes. Autophagosomes are typically bilayer structures containing undigested cytoplasmic components or organelles (such as mitochondria and endoplasmic reticulum fragments) that do not fuse with lysosomes. When autophagosomes are fused into autophagolysosomes, they become monolayers containing cytoplasmic components. Observation by TEM could merely prove the existence of autophagy, and it is difficult to reflect intensity of autophagy activity only by the number of autophagy structures, which is random to some extent. Therefore, it is necessary to combine with other detection methods for further analysis. In this study, no autophagosome was found in NC rats. There was an autophagosome in the DGP group. One autophagosome and two autophagolysosomes were observed in the EA group. Two phagophores formed by endoplasmic reticulum (ER) were observed in the MP group, extending and wrapping mitochondrion, marking the beginning of autophagy. Membrane structures of two phagophores in the MP group were found from ER. Besides, most of the autophagosome recognizable contents were mitochondria.

LC3 is involved in the formation of autophagosomal membranes, including two mutually convertible forms, LC3 I and LC3 II. LC3 I is modified by ubiquitination and then combined with PE on the membrane of autophagosomes, turning into membrane-binding LC3 II. LC3 II is located in preautophagosomes and autophagosomes, the symbol of autophagosomes, which increases with the increase of autophagosomes' membrane [26]. P62, also known as SQSTM1, connects LC3 and ubiquitinized substrates, which are then integrated into autophagosomes and eventually degraded in autophagosomes. When autophagy is induced, autophagosomes fuse with lysosomes, and P62 is degraded by lysosomal enzymes. If the fusion dysfunction of autophagosomes and lysosomes happens, P62 cannot be degraded by lysosomes, and P62 will be accumulated and the amount of P62 would be increased. In this study, the ratios of LC3 II/LC3 I and P62 were detected through Western blotting. According to Figure 8, LC3 II/LC3 I of the DGP group was much higher than the NC group, and the EA group was higher than the MP group. The expression of P62 protein in the DGP group was the highest, and the second was the MP and EA groups, the NC group least. The trend of LC3 II/LC3 I in the four groups was completely consistent with the trend of P62. That is to say, both LC3 II/LC3 I and P62 in DGP rats were significantly increased. It indicated that fusion of autophagosomes and lysosomes in ICCs was blocked. And EA and MP effectively relieved the impaired autophagy flux.

The results of this experiment are consistent with relevant studies. Fang et al. found that apelin inhibited autophagy in podocytes through ERK/Akt/mTOR-dependent pathways, which leads to podocyte apoptosis and diabetic kidney disease [27]. Liu et al. found inhibited autophagy in podocytes of diabetic patients promoted the progression of diabetic nephropathy through ERK, Akt, and mTOR pathway [28]. But Aysa Rezabakhsh also found human mesenchymal stem cells upregulated autophagy in serum of type II diabetes [29]. This difference with this study may be related to the following factors. The regulatory mechanism of diabetic rats was more complex than cells cultured in serum of type 2 diabetes, and the hyperglycemia time (8 weeks) in the rat model was much longer than that in cell culture (7 days).

In conclusion, the suppression of autophagy in ICCs is the pathological basis of diabetic gastroparesis, and EA could ease the disorder of autophagy to improve gastric motility.

## Data Availability

The datasets used and/or analyzed during the current study are available from the corresponding author on reasonable request.

## References

[1] Koch K. L., Calles-Escandón J. (2015). Diabetic gastroparesis. *Gastroenterology Clinics of North America*.

[2] Vanormelingen C., Tack J., Andrews C. N. (2013). Diabetic gastroparesis. *British Medical Bulletin*.

[3] Bytzer P., Talley N. J., Leemon M., Young L. J., Jones M. P., Horowitz M. (2001). Prevalence of gastrointestinal symptoms associated with diabetes mellitus. *Archives of Internal Medicine*.

[4] Hasler W. L. (2012). Gastroparesis. *Current Opinion in Gastroenterology*.

[5] Shin D. H., Lee M. J., Jiao H. Y. (2015). Regulatory roles of endogenous mitogen-activated protein kinases and tyrosine kinases in the pacemaker activity of colonic interstitial cells of cajal. *Pharmacology*.

[6] Takaki M. (2003). Gut pacemaker cells: the interstitial cells of Cajal (ICC). *Journal of Smooth Muscle Research*.

[7] Farrugia G. (2008). Interstitial cells of Cajal in health and disease. *Neurogastroenterology & Motility*.

[8] Grover M., Farrugia G., Lurken M. S. (2011). Cellular changes in diabetic and idiopathic gastroparesis. *Gastroenterology*.

[9] Lin Z., Sarosiek I., Forster J., Damjanov I., Hou Q. (2010). Association of the status of interstitial cells of Cajal and electrogastrogram parameters, gastric emptying and symptoms in patients with gastroparesis. *Neurogastroenterol Motil*.

[10] Lin G., Zhang J., Li L. (2016). Effect of electroacupuncture on gastric interstitial cells of Cajal in a rat model of diabetic gastroparesis. *Experimental and Therapeutic Medicine*.

[11] Levine B., Klionsky D. J. (2004). Development by self-digestion. *Developmental Cell*.

[12] Kobayashi S., Xu X., Chen K., Liang Q. (2012). Suppression of autophagy is protective in high glucose-induced cardiomyocyte injury. *Autophagy*.

[13] Yj Z. (2013). Diagnosis and treatment of diabetic gastroparesis. *Medical Recapitulate*.

[14] Zhang Xuyin X. H. (2016). Clinical observation of electroacupuncture in the treatment of functional dyspepsia. *Shanghai Journal of Acupuncture and Moxibustion*.

[15] XD M. (2017). Acupuncture and moxibustion for postoperative gastric motility disorders in 129 cases. *Clinical Journal of Chinese Medicine*.

[16] Peng Y., He F., Wan Q. (2016). Effect of electroacupuncture on Ghrelin and GHSR protein and gene expression in gastric antrum of diabetic gastroparesis rats. *Chinese Journal of Basic Medicine in Traditional Chinese Medicine*.

[17] Lin Yaping W. Q., Peng Y. (2015). The effects of electroacupuncture on the expressions of ghrelin mRNA and ghrelin receptor mRNA in gastric antrum of diabetic gastroparesis rats. *Acupuncture Research*.

[18] Zhang C. C., Lin Y. P., Peng Y., Yue Z. H., Chen H. J. (2017). Effects of electroacupuncture on ultrastructure of interstitial cells of cajal and stem cell factor-kit signal pathway of gastric antrum in diabetic gastroparesis rats. *Acupuncture Research*.

[19] Chen Haijiao L. Y. (2016). Effect of electricity on gastric emptying rate and electrogastrogram in diabetic gastroparesis rats. *Journal of Traditional Chinese Medicine University of Hunan*.

[20] Chengcheng Z. (2018). *Effect of Electroacupuncture on Pacing Function of Cajal Stromal Cells in Diabetic Gastroparesis Rats*.

[21] S. G. Y. Yu, 2009, Experimental acupuncture

[22] Chen Y., Xu J. J., Liu S., Hou X. H. (2013). Electroacupuncture at ST36 ameliorates gastric emptying and rescues networks of interstitial cells of Cajal in the stomach of diabetic rats. *PLoS One*.

[23] Wu X. F., Chen X. L., Zheng X. N., Guo X., Xie Z. Q. (2018). Effect of different stimulating strength of electroacupuncture on gastrointestinal motility and RhoA/ROCK signaling in gastric antral smooth muscle in diabetic gastroparesis rats. *Acupuncture Research*.

[24] Zhang C. C., Lin Y. P., Peng Y., Yue Z. H., Chen H. J. (2017). Effects of electroacupuncture on ultrastructure of interstitial cells of cajal and stem cell factor-kit signal pathway of gastric antrum in diabetic gastroparesis rats. *Acupuncture Research*.

[25] Matboli M., Eissa S., Ibrahim D., Hegazy M. G. A., Imam S. S. (2017). Caffeic acid attenuates diabetic kidney disease via modulation of autophagy in a high-fat diet/streptozotocin- induced diabetic rat. *Scientific Reports*.

[26] Schaaf M. B. E., Keulers T. G., Vooijs M. A., Rouschop K. M. A. (2016). LC3/GABARAP family proteins: autophagy-(un) related functions. *The FASEB Journal*.

[27] Fang L., Zhou Y., Cao H. (2013). Autophagy attenuates diabetic glomerular damage through protection of hyperglycemia-induced podocyte injury. *PLoS One*.

[28] Liu Y., Zhang J., Wang Y. (2017). Apelin involved in progression of diabetic nephropathy by inhibiting autophagy in podocytes. *Cell Death and Disease*.

[29] Rezabakhsh A., Cheraghi O., Hassanpour A. (2017). Type 2 diabetes inhibited human mesenchymal stem cells angiogenic response by over-activity of the autophagic pathway. *Journal of Cellular Biochemistry*.

